# *Gardnerella* *vaginalis*-binding IgA in the urethra of sexually experienced males

**DOI:** 10.1186/s40168-024-02007-4

**Published:** 2025-01-29

**Authors:** Rachel Liu, R. M. Galiwango, Daniel Park, Sanja Huibner, Maliha Aziz, Aggrey Anok, James Nnamutete, Yahaya Isbirye, John Bosco Wasswa, Deo Male, Godfrey Kigozi, Aaron A. R. Tobian, Jessica L. Prodger, Cindy Liu, Bryan Coburn, Rupert Kaul

**Affiliations:** 1 Department of Medicine, University of Toronto, Toronto, Canada; 2 Department of Immunology, University of Toronto, Toronto, Canada; 3 Rakai Health Science Program, Kalisizo, Uganda; 4 Department of Environmental and Occupational Health, Milken Institute School of Public Health, George Washington University, Washington, DC USA; 5 Department of Pathology, John Hopkins University School of Medicine, Baltimore, MD USA; 6 Department of Microbiology and Immunology, Western University, London, ON Canada; 7 Toronto General Hospital Research Institute, University Health Network, Toronto, ON Canada

**Keywords:** Genital microbiome, Genital immunology, Male genital tract, Mucosal antibodies, Urethral microbiome, Urethral secretions

## Abstract

**Background:**

Genital inflammation increases HIV susceptibility and is associated with the density of pro-inflammatory anaerobes in the vagina and coronal sulcus. The penile urethra is a critical site of HIV acquisition, although correlates of urethral HIV acquisition are largely unknown. While *Streptococcus mitis* is a consistent component of the urethral flora, the presence of *Gardnerella vaginalis* has been linked with prior penile-vaginal sex and urethral inflammation. Here, we use a flow cytometry-based bacterial assay to quantify urethral IgA and IgG that bind *G. vaginalis* and *S. mitis* in a cross-sectional cohort of 45 uncircumcised Ugandan men and to evaluate their association with the urethral microbiome and local soluble immune factors.

**Results:**

Urethral antibodies binding both bacterial species were readily detectable, with *G. vaginalis* predominantly bound by IgA, and *S. mitis* equivalently by IgA and IgG. *Gardnerella vaginalis*-binding IgA was elevated in participants with detectable urethral *Gardnerella*, with the latter only present in participants who reported prior penile-vaginal sex. In contrast, detectable urethral *S. mitis* was not associated with sexual history or levels of *S. mitis-*binding IgA/IgG. The time from the last penile-vaginal sex was inversely correlated with the urethral concentrations of total IgA, *G. vaginalis*-binding IgA, and chemokines IL-8 and MIP-1β; these inflammatory chemokines were independently associated with higher total IgA concentration, but not with *G. vaginalis-*binding IgA.

**Conclusions:**

This first description of microbe-binding antibodies in the penile urethra suggests that urethral colonization by *Gardnerella* after penile-vaginal sex specifically induces a *G. vaginalis*-binding IgA response. Prospective studies of the host-microbe relationship in the urethra may have implications for the development of vaccines against sexually-transmitted bacteria.

Video Abstract

**Supplementary Information:**

The online version contains supplementary material available at 10.1186/s40168-024-02007-4.

## Background

Globally, penile-vaginal sex is the most common route of HIV transmission [1]. Penile HIV acquisition primarily occurs in either the foreskin or the distal urethra [2, 3]; the latter is thought to be the predominant tissue site of penile HIV acquisition for circumcised men and approximately a third of uncircumcised men. In keeping with this, the urethra harbors several HIV target cell subsets, including CD4 + T cells and CD4 + CCR5 + macrophages throughout the urethra [2] and CD1a + dendritic cells in the meatus and fossa navicularis [4]. HIV susceptibility in the coronal sulcus is enhanced by inflammation, either induced by sexually transmitted infections or by inflammatory components of the bacterial microbiome [5, 6]. However, the immune and microbial determinants of HIV susceptibility in the distal urethra are much less understood.

Although direct links between urethral HIV acquisition and the local microbiome and immune milieu have not been established, both parameters modulate HIV susceptibility in the penile coronal sulcus and the female genital tract (FGT). In the coronal sulcus, the density of six Bacteria Associated with Seroconversion, Inflammation, and Immune Cells (BASIC) species is linked to increased HIV susceptibility, due to their ability to induce pro-inflammatory cytokines and chemotactic chemokines such as IL-8 and thus to recruit HIV-susceptible cells to foreskin tissues [5, 7]. BASIC species include *Peptostreptococcus anaerobius*, *Prevotella bivia*, *Prevotella disiens*, *Dialister propionicifaciens*,* Dialister micraerophilus*, and a genetic near neighbor of *Dialister succinatiphilus*; however, they are less abundant and less inflammatory in the urethra [8]. Instead, Toh et al. identified two community clusters within the microbiome of the penile urethra, one dominated by *Streptococcus mitis*, and the other by *Gardnerella vaginalis* [9]. The latter is strongly associated with penile-vaginal sex [9], inflammation in both the urethra and the FGT [10, 11], and HIV acquisition in women [12], while *S. mitis* is not associated with penile inflammation [7].

The penile urethra contains abundant antibody-producing plasma cells [13, 14]; these may induce antibody responses to the local microbiota that can modulate the local microbial composition, the local immune milieu, and HIV susceptibility. In the gut, mucosal antibodies—particularly IgA—are induced by the local microbiota and can shape microbial composition and modulate host-microbe interactions through mechanisms that include receptor blockage, neutralization, immune exclusion, and complement activation [15–19]. Recent studies in the FGT found that total vaginal levels of IgA and IgG, as well as microbe-binding IgA and IgG that bound key vaginal bacteria, were each inversely correlated with bacterial load and positively correlated with multiple inflammatory cytokines and chemokines previously linked to HIV acquisition [20, 21]. It is unknown whether microbe-binding antibodies are present in the urethra or if they play a role in regulating the local microbiome and inflammation in this tissue.

Here we use a flow cytometry-based assay to quantify IgA and IgG from the distal penile urethra of uncircumcised men that bind *G. vaginalis* and *S. mitis* and to investigate associations with the urethral microbiota and local immune milieu. Specifically, we hypothesized that *G. vaginalis-*binding IgA and IgG would be associated with sexually acquired *Gardnerella*, bacterial clearance, and the induction of pro-inflammatory cytokines and chemokines.

## Methods

### Study enrollment and behavioral data

The study protocol was reviewed and approved by the Research and Ethics Committee (REC) of the Uganda Virus Research Institute in Entebbe (Uganda) and the Institutional Review Board at the University of Toronto (Canada). All study participants gave written informed consent. Study participants consisted of uncircumcised HIV-uninfected Ugandan men who did not have any genital STI symptoms, aged at least 18 years old, and electively presenting at the Rakai Health Sciences Program for voluntary medical male circumcision to reduce HIV risk as previously described [8]. Urine samples were collected and tested for *Neisseria gonorrhoeae* and *Chlamydia trachomatis,* and treatment was provided as needed according to Uganda National Guidelines. A social-behavioral questionnaire was administered that included a detailed sexual history. In addition to other socio-behavioral questions, participants indicated whether they had ever engaged in penile-vaginal, oral, or anal sex. Of the 51 uncircumcised male participants enrolled in the original cohort, only 45 participants had available urethral supernatant from the baseline visit and were included in this sub-study.

### Sample collection and processing

A nylon flocked swab (Hardy Diagnostics, CA, USA) was inserted in the distal urethra and rotated by the study clinician before being placed into 500 μL of PBS and transported to the laboratory on ice. Swabs were vigorously vortexed for 60 s in the laboratory, before inverting the swab within the tube and performing a quick spin to dry out the swab. Swabs were then discarded and the eluants were stored at − 80 °C for further analysis.

### Soluble immune factor measurement

Urethral swabs were thawed and re-centrifuged at 2000 rpm for 5 min. The following soluble immune factors were assayed: IL-1α, IL-1β, IL-8, MIP-1β, soluble E-cadherin (sE-cad), MMP-9, TIMP-1, and VEGF in duplicate by Multiplex MSD according to the manufacturer’s instructions (Meso Scale Discovery, Rockville, MD). Soluble E-cadherin is a marker of epithelial disruption in the genital tract, and MMP-9 induces epithelial disruption [22, 23]. A standard curve was generated using serial dilutions and was used to determine the lower and upper limit of detection, and the concentration of each analyte (pg/mL). The LLOQ was calculated across all plates as the mean of the LLOD plus one standard deviation. Values below the LLOQ were assigned the LLOQ value, and values above the ULOQ were assigned the ULOQ value, regardless of their coefficient of variation. A frozen control media aliquot was included on each plate to monitor inter-plate/run variability.

### Microbiome analysis

Using a combination of enzymatic and chemical lysis, DNA was extracted from 80 μL of diluted swab eluent. At 37 °C and for 1 h, each sample was treated with an enzymatic cocktail containing 122 μL Tris–EDTA, 50μL 10 mg/mL lysozyme (L6876-1G, Sigma-Aldrich), 4 μL 25 KU/mL mutanolysin (M4782-5KU, Sigma-Aldrich), and 3 μL 4 U/μL lysostaphin (SAE0091-2MG, Sigma-Aldrich) followed by extraction using MagMax DNA Multi-Sample Ultra 2.0 Kit (including Proteinase K treatment) with 80 μL final elution volume. A negative extraction control (NEC) and a positive extraction control (PEC) were included in each extraction batch. Penile bacterial communities and all extraction controls were assessed using 16S rRNA gene-based sequencing and broad-range real-time PCR (cite BacqtQuant). NEC Cp > 33–35 were considered acceptable. NEC sequencing results were further analyzed for potential sequencing reagent contamination. PEC sequencing results were analyzed for consistency in extraction efficiency and sequencing performance. A modified protocol from Fadrosh [24] with forward (341F) and reverse (786R) primers from Liu [25] was used to perform the sequencing analysis. A no-template control (NTC) was included in each PCR plate and analyzed by gel electrophoresis for PCR reagent contamination, and a positive template control (PTC) was included to confirm PCR reagent performance. Using MiSeq Reagent Kit v3 (600 cycles), sequencing was performed on the MiSeq platform. Using cutadapt v2.4, primer sequences were removed during processing, and Trimmomatic v0.39 was used to quality-trim the resultant sequences. For reads-filtering, chimera check, and inferred error models to identify amplicon sequence variants (ASVs), DADA2 v1.10 modules were used. The Naïve Bayesian Classifier (v.2.12) was used to classify the ASVs at each taxonomic level at an 80% bootstrap confidence level. To generate an abundance matrix for analysis, classification results for each sample were enumerated. Additional details can be found at https://github.com/araclab/mb_analysis. Taxa were delineated only to the genus level. The estimated absolute abundance of each taxon was calculated using the total bacterial load per swab (total 16S rRNA copies) multiplied by the proportional relative abundance (%). The sequencing read achieve project (SRA) number PRJNA738496 can be used to access sequence data for this study.

### Total IgA and IgG quantification

Urethral swab supernatants were diluted 1/100 in PBS with 1% Tween 20 and 10% BSA and centrifuged at 10,000 g for 10 min. Total IgA and IgG levels were quantified using human IgA and IgG (total) uncoated ELISA kits (Invitrogen) according to the manufacturer’s instructions. The LLOQ was determined using the lowest value generated from the standard curve of each plate. Only samples above the ULOQ were diluted and re-analyzed. Internal laboratory controls were used as inter-assay controls.

### Bacterial preparation

*Gardnerella* *vaginalis* (ATCC, 14019) was grown in New York City III agar anaerobically (80% N_2_; 10% CO_2_; 10 H_2_) at 37 °C for 46–48 h. *Streptococcus mitis* (NCTC 12261 [NS51]) was grown in Brain Heart Infusion broth aerobically (5% CO_2_) at 37 °C for 18–20 h. Bacteria were harvested at the concentration of ~ 1 × 10^9^ CFU and stored as frozen glycerol (20%) aliquots before usage. Bacteria were stained with CFSE (Thermo Fischer) for 30 min and washed before incubation with clinical samples.

### Microbe-binding assay

The anti-commensal antibody protocol was adapted from Moor et al. [26]. Urethral supernatants were heat-inactivated for 30 min at 56 °C and serially diluted in FACs buffer (1:2 dilutions in FACs [2% BSA, 5uM EDTA-PBS]). Equal volumes of urethral samples were combined with an equal volume of bacteria (~ 10^5^ CFU/mL) and incubated for 1 h at 4 °C. Antibody-bound bacteria were stained with anti-human goat IgG F(ab’)2 antibody (AF594, Jackson Immunology Inc.) and anti-human goat IgA F(ab’)2 antibody (AF647, Southern Biotech). All samples were analyzed using an LSR Fortessa (Becton Dickinson) with settings optimized for bacterial cell detection.

### Flow cytometry analysis

Flow cytometry analysis of samples was performed using FlowJo (Treestart Inc.), representative gating strategies previously described [21]. Geometric mean fluorescence intensity (MFI) values were extracted for each sample at each dilution. Bacteria incubated without clinical samples and stained with secondary antibodies were used as the negative control and were subsequently subtracted from all samples. Sample dilution factors and their corresponding MFI values were log10 transformed and plotted with a linear regression using GraphPad prism to produce regression lines for each sample. Negative log10 transformed values were assigned a zero value. Responses were reported as the area under the curve (AUC) value for each regression line. IgG + and IgA + events were reported from the highest concentration dilution of each urethral sample.

### Statistical analysis

Analysis was performed using GraphPad Prism (version 10.1.0) and RStudio (version 2022.07.02). Soluble immune factors, estimated absolute abundance, and antibody concentrations were log10 transformed for all analyses. *G. vaginalis*-binding IgA and IgG, total IgA and IgG, and soluble immune factors were compared between groups using either Welch’s *T* test or the Mann–Whitney *U* test. Comparisons within the group used the Wilcoxon test for paired analysis and the one-sample Wilcoxon test when testing against a hypothetical mean. Heatmaps were created in R using the package heatmap.2. Antibody proportion responses were assessed using with Wilcoxon test or Kruskal–Wallis test with multiple comparisons. Correlations of time since the last vaginal sex were assessed using Spearman’s correlation. Multiple linear regression was used to control for the effects of antibodies and *G. vaginalis* abundance when assessing associations with soluble immune factors. Mediation analysis was conducted in R and bootstrapped 500 times using the mediation package.

## Results

### Participant characteristics

A total of 45 participants were included in this sub-study. The median age of participants was 20 years (Table 1), and 41 (91.1%) reported a prior history of penile-vaginal sex. Participants with a history of penile-vaginal sex reported a median lifetime number of four sexual partners and 33/41 (80.4%) reported penile-vaginal sex within the 6 months prior to sampling. The median time from the last penile-vaginal sex was ~ 60 days. Antibiotic use in the past 3 months was uncommon (4.4%). While no participants had symptoms of dysuria or urethral discharge, two participants (4.4%) tested positive for asymptomatic infection by *Chlamydia trachomatis* and were included in all subsequent analyses.

**Table 1:** Participant characteristics (*n* = 45). Values are reported as either count (percentage) or as median (IQR)

Characteristic	Total (*n* = 45)
Age	20 (18–24)
Ever had penile-vaginal sex	
Yes	41 (91.1%)
No	4 (8.9%)
Current marital status	
Married/cohabiting	6 (13.3%)
Divorced/separated	2 (4.4%)
Never married	37 (82.2%)
Number of lifetime sexual partners	4 (2–10)
Number of sexual partners in last 6 months	1 (0–2)
Number of days since last penile-vaginal sex (of those participants reporting prior penile-vaginal sex)	60 (10.5–120)
Antibiotic use in the past 3 months	2 (4.4%)

### Total urethral IgA and IgG levels

**Fig. 1:**
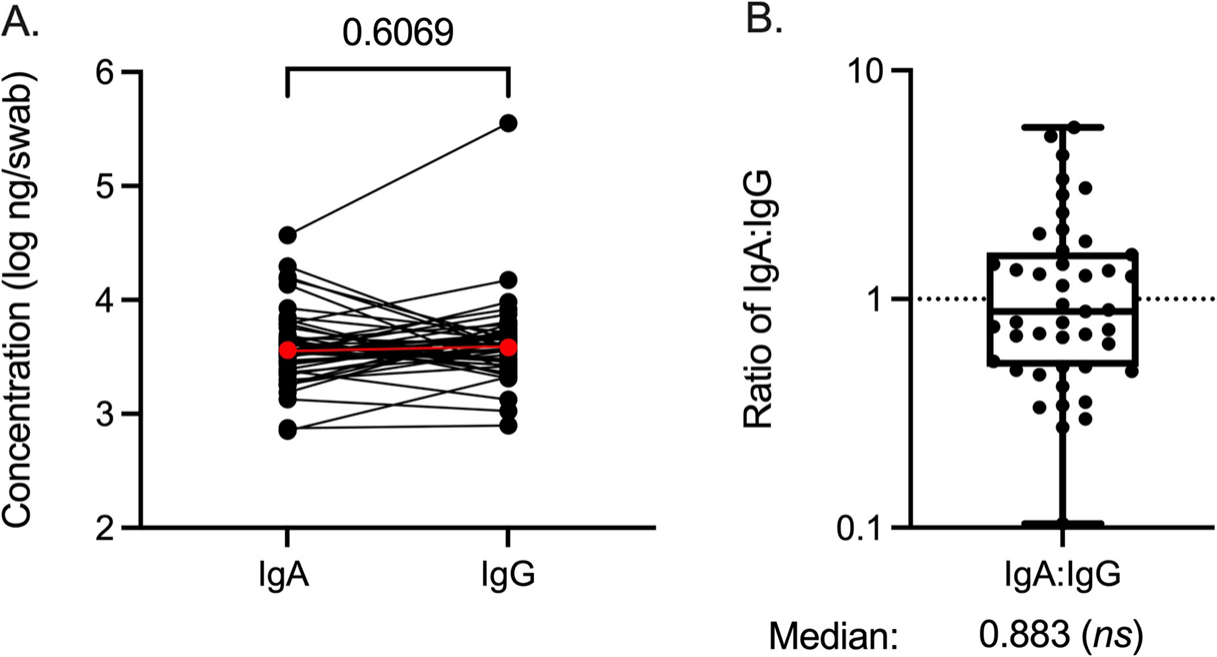
Total IgA and IgG concentrations in distal urethral swabs. **A** Log_10_ concentrations of paired IgA and IgG from the urethra, with the median value shown in red. **B** The ratio of IgA to IgG concentrations within the cohort (both *n* = 45). Statistical analysis used the Wilcoxon test and one-sample Wilcoxon test against a hypothetical IgA to IgG ratio of 1

We quantified total IgA and IgG concentrations in urethral swab eluents; all participants had quantifiable antibody concentrations above the LLOQ. The median concentration of IgA was 3615 ng/mL (IQR: 2423–5315), and the median concentration of IgG was 3815 (IQR: 2671–5120.5) ng/mL, with no significant difference between IgA and IgG concentrations (Fig. 1A; = 0.607). Neither IgA nor IgG concentrations were correlated with the number of lifetime sexual partners or a history of prior penile-vaginal sex. However, total IgA concentration was correlated with a higher number of recent female sexual partners (within the past 6 months; *r* = 0.342, *p* = 0.021) and with a lower number of days since last vaginal sex (*r* = − 0.411, *p* = 0.005). Total urethral IgG concentration was not correlated with the number of female sexual partners (*r* = − 0.044, *p* = 0.783) or days since the last vaginal sex (*r* = 0.128, *p* = 0.403).

### Microbe-binding IgA and IgG in urethral swabs

**Fig. 2:**
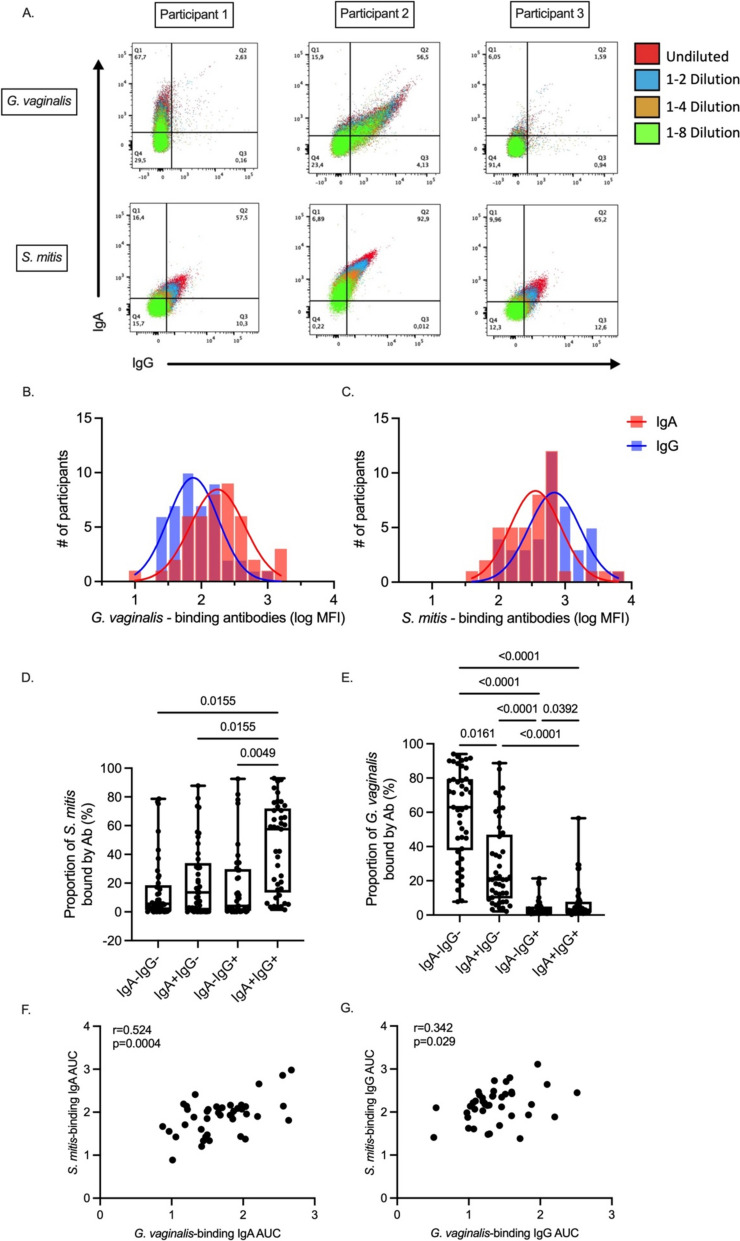
Microbe-binding IgA and IgG within distal urethral swabs. **A** Representative flow cytometry plots of IgA and IgG MFI responses to *G. vaginalis* and *S. mitis*. The figure legend on the right illustrates the sample dilutions, with red indicating the most concentrated dilution and green the least concentrated dilution. Histogram distribution of the log_10_ transformed IgA and IgG MFI responses at the highest dilution concentration to **B ***G. vaginalis* (*n* = 45) and **C ***S. mitis* (*n* = 41)*.* A comparison of the **D**–**E** proportion of double negative (IgA − IgG −), single positive (IgA + IgG − , IgA − IgG +), and double positive (IgA + IgG +) bacterial populations. Correlation between **F ***S. mitis*-binding IgA AUC vs. *G. vaginalis*-binding IgA AUC and **G ***S. mitis*-binding IgG AUC vs. *G. vaginalis*-binding IgG AUC. Statistical analysis utilized the Wilcoxon test, Kruskal–Wallis test, and Spearman correlation

Next, we quantified the ability of urethra-derived IgA and IgG to bind *Gardnerella vaginalis* and *Streptococcus mitis* using a flow cytometry-based assay (Fig. 2A). *G. vaginalis*-binding antibodies were assessed in all 45 participants; however, due to the limited sample volumes, we were only able to assess *S. mitis*-binding antibody responses in 41 participants. The four participants with insufficient samples did not differ in their participant characteristics from the 41 analyzed.

Four serial dilutions of urethral swab supernatant were incubated separately with *G. vaginalis* and *S. mitis*, stained with fluorophore-labeled secondary antibodies, and analyzed by flow cytometry (Materials and Methods). All analyzed samples had MFI values above zero in their highest concentration dilution after subtracting for background signals measured in the negative control. The mean IgA AUC values were 1.687 ± 0.441 for *G. vaginalis* and 1.900 ± 0.423 for *S. mitis,* and the mean IgG AUC values were 1.364 ± 0.397 for *G. vaginalis* and 2.167 ± 0.392 for *S. mitis.* Although *G. vaginalis*-binding antibodies were detected in most urethral samples, approximately two-thirds of all lab-cultured *G. vaginalis* remained unbound by either urethral IgA or IgG (IgA − IgG − proportion: 62.39 (IQR: 37.90–79.38)%). When present, *G. vaginalis*-binding antibodies were predominantly IgA (IgA + proportion: 27.00% (IQR: 16.75–52.70%), IgG + proportion: 5.40% (IQR: 3.51–12.05%)), and most *G. vaginalis* bound by urethra IgA were not additionally bound by urethral IgG (i.e., IgA + IgG − ; Fig. 2D). In contrast, antibody binding to *S. mitis* was mediated by IgA and IgG in equivalent proportion, with most lab-cultured *S. mitis* demonstrating double binding (IgA + IgG + median proportion: 57.50% (IQR: 13.45–71.95%), Fig. 2E). *Gardnerella vaginalis* and *S. mitis*-binding IgA AUC were positively correlated with each other (*r* = 0.524, *p* = 0.004, Fig. 2F) and total urethral IgA concentration (*G. vaginalis-*binding IgA:* r* = 0.381, *p* = 0.010; *S. mitis*-binding IgA*: r* = 0.498, *p* < 0.001). Similarly, *S. mitis*-binding IgG and *G. vaginalis*-binding IgG AUCs were correlated with each other (*r* = 0.324, *p* = 0.020, Fig. 2G), but only *S. mitis*-binding IgG was positively correlated with the total urethral IgG concentration (*G. vaginalis*-binding IgG: *r* = 0.204, *p* = 0.178; *S. mitis*-binding IgG: *r* = 0.539, *p* < 0.001).

### Microbe-binding IgA and IgG and urethra bacterial abundance

Next, we investigated whether the presence and/or density of urethral microbes correlated with microbe-binding IgA and IgG. Within the cohort, 18 participants (40%) had detectable *Gardnerella* in the urethra (Supplementary Fig. 1), all of whom reported prior penile-vaginal sex. Conversely, the majority of participants (*n* = 37, 82.2%) had detectable urethral *Streptococcus*, and did not differ based on prior penile-vaginal sex. Over 97% of the *Gardnerella* detected by 16S rRNA sequencing were classified as *G. vaginalis,* and over 94% of the *Streptococcus* species analyzed belong to the *Streptococcus mitis* group, which is a cluster of 13 closely related species within the genus *Streptococcus* which includes *S. mitis* [27].

**Fig. 3:**
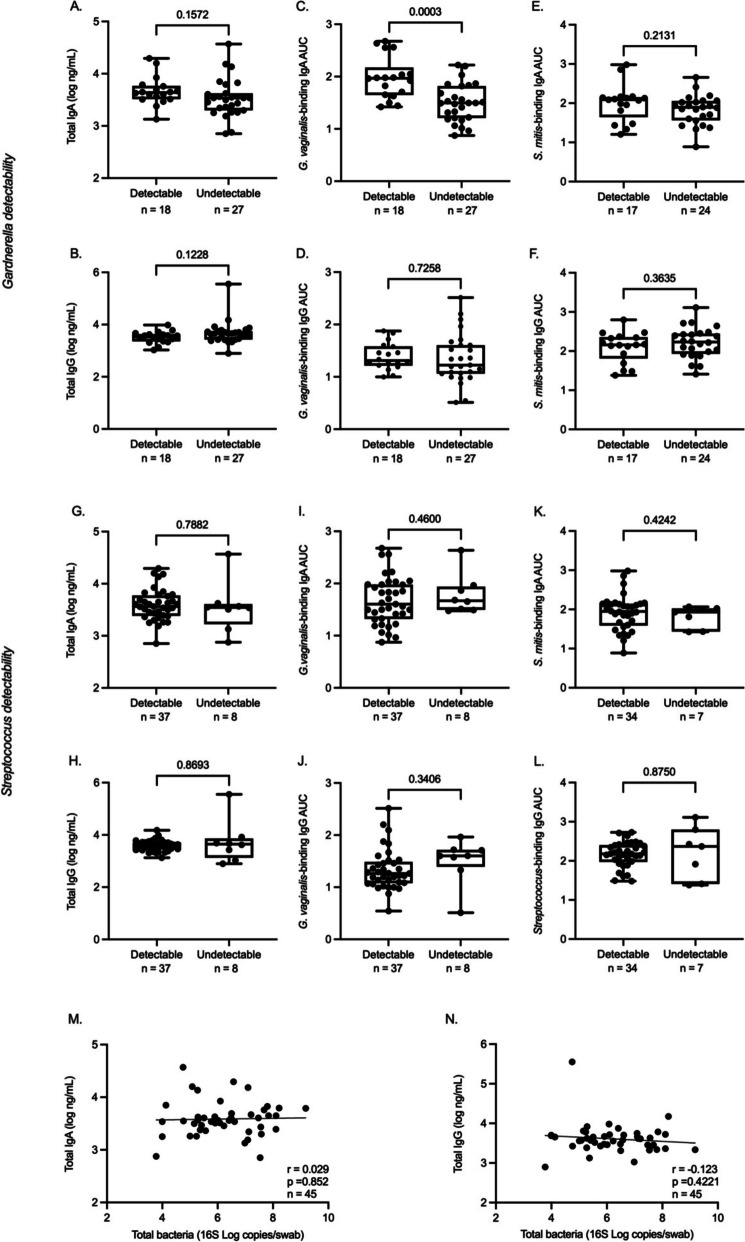
Associations between urethral IgA and IgG and the urethral microbiome. Comparison of total IgA and IgG concentrations, *G. vaginalis*-binding IgA and IgG, and *S. mitis*-binding IgA and IgG between participants with and without detectable levels of urethral **A**–**F** *Gardnerella* and **G**–**L** *Streptococcus.* Correlations between total bacterial abundance with **M** total IgA concentration and **N** total IgG concentration. Statistical analysis utilized the Welch’s *T*-test, Mann–Whitney *U* test, and Spearman’s correlation

Participants with detectable urethral *Gardnerella* had similar total concentrations of IgA and IgG in the urethra compared to those without detectable *Gardnerella* (Fig. 3A–B; IgA: *p* = 0.157, IgG: *p* = 0.123); however, they exhibited elevated *G. vaginalis*-binding IgA AUCs (Fig. 3C; mean difference: − 0.476 ± 0.118, *p* = 0.0003). Among participants with detectable *Gardnerella,* no correlation was apparent between the estimated absolute abundance of *Gardnerella* and *G. vaginalis-*binding IgA AUC response (*r* = 0.139, *p* = 0.581). In contrast, neither the AUC of *S. mitis-*binding IgA nor IgG was associated with the presence (Fig. 3K–L) or estimated abundance of *Streptococcus* (*S. mitis*-binding IgA: *r* = 0.129, *p* = 0.265; *S. mitis*-binding IgG: *r* = 0.2188, *p* = 0.217).

Total bacterial density in the urethra, as assessed by 16S qPCR, was not correlated with either total IgA or IgG concentrations (Fig. 3M–N), *S. mitis-*binding IgA or IgG, or *G. vaginalis-*binding IgG. However, *G. vaginalis-*binding IgA did correlate with elevated total urethral bacterial density (*r* = 0.313, *p* = 0.036). These results generally remained unchanged when excluding the two participants with asymptomatic *Chlamydia trachomatis* infection, except those participants with detectable *Streptococcus* (*n* = 36) had lower *G. vaginalis-*binding IgG response than participants with undetectable *Streptococcus* (*n* = 7; difference of means: 0.312 ± 0.097, *p* = 0.005).

### Prior penile-vaginal sex and urethral IgA and IgG

*Gardnerella* was only present in the urethra of participants with a self-reported history of penile-vaginal sex, suggesting sexual acquisition [10]. Importantly, we did not find any additional taxa in the urethral microbiota that were associated with a prior history of penile-vaginal sex. We compared urethral total antibody levels and microbe-binding antibodies between participants who reported no prior history of penile-vaginal sex (*n* = 4) and those who reported a prior history of penile-vaginal sex (*n* = 41). Total levels of urethral IgA and IgG did not differ between groups, but *G. vaginalis-*binding IgA was significantly higher in participants with a prior history of penile-vaginal sex (mean AUC difference: 0.4009 ± 0.1157, *p* = 0.0096). *S. mitis*-binding IgA and IgG did not differ based on prior history of penile-vaginal sex, although we were only able to assess *S. mitis*-binding antibody responses in three of the four participants who did not report prior penile-vaginal sex.

**Fig. 4:**
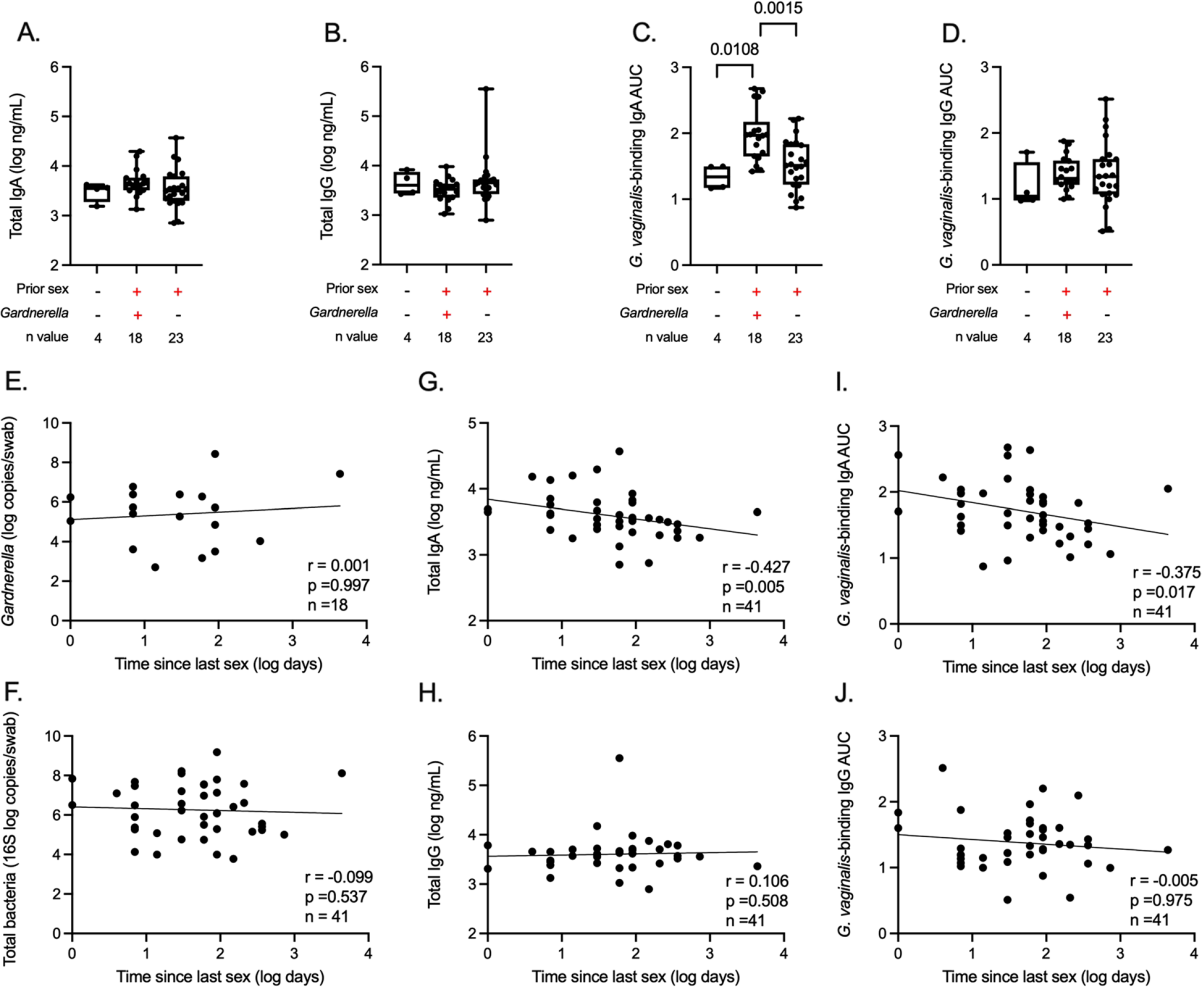
Prior penile-vaginal sex and urethral IgA and IgG. **A** Total IgA and **B** IgG concentrations, **C** *G. vaginalis*-binding IgA AUC, **D** and *G. vaginalis*-binding IgG AUC were compared between study participants who reported: no prior history of sex, prior history of sex with detectable urethral *Gardnerella*, and prior history of sex without detectable urethral *Gardnerella*. Correlations between time since last penile-vaginal sex with **E** *Gardnerella* relative abundance among participants with detectable urethral *Gardnerella*, **F** total urethral bacterial abundance, **G** total IgA and **H** IgG concentration, **I** *Gardnerella*-binding IgA AUC, and **J** *Gardnerella*-binding IgG AUC. Only participants with a prior history of penile-vaginal sex were included in the correlations. The statistical analysis utilized the Kruskal–Wallis test, one-way ANOVAs, and Spearman’s correlation

Subsequently, we stratified participants into three groups: participants reporting no prior penile-vaginal sex (*n* = 4), participants with a prior history of penile-vaginal sex and detectable urethral levels of *Gardnerella* (*n* = 18)*,* and participants with a prior history of penile-vaginal sex and no detectable urethral *Gardnerella *(*n* = 23)*.* Participants with a prior history of sex and detectable levels of *Gardnerella* had elevated *G. vaginalis* binding IgA AUC when compared to those participants who reported never engaging in penile-vaginal sex and those participants with a prior history of penile-vaginal sex but no detectable *Gardnerella* (mean AUC difference to participants with no prior history of sex: 0.6371, *p* = 0.011; mean AUC difference to participants with a prior history of sex but no detectable *Gardnerella*: 0.439, *p* = 0.002; Fig. 4C). The AUC of *G. vaginalis*-binding IgA in the urethra was similar in sexually inexperienced participants and those with prior sexual experience but no detectable levels of *Gardnerella* (mean AUC difference: 0.198, *p* = 0.600).

Next, we assessed whether the time elapsed since the last reported vaginal sex was associated with *Gardnerella* abundance, *G. vaginalis-*binding IgA and IgG, or total IgA and IgG concentrations. While the detection (prevalence) of urethral *Gardnerella* was associated with a shorter duration since the last vaginal sex (log_10_ difference in days since last sex = 0.477, *p* = 0.020), the estimated absolute abundance of *Gardnerella* among these participants was not correlated with time since the last sex (Fig. 4E; *r* = 0.001, *p* = 0.997). The number of days since last vaginal sex was also not correlated with total bacterial abundance in the urethra (Fig. 4F; total bacterial abundance: *r* = − 0.099), but was strongly correlated with the total urethral IgA concentration (Fig. 4G; *r* = − 0.427, *p* = 0.005) and *G. vaginalis-*binding IgA (Fig. 4I; *r* = − 0.375, *p* = 0.017). These findings remained unchanged when excluding the two participants with urethral chlamydia (data not shown).

### Soluble immune factors and urethral microbe-binding antibodies

We analyzed associations between urethral soluble immune factors and urethral IgA and IgG, hypothesizing that both total IgA and IgG concentration and microbe-binding IgA and IgG would be associated with increased pro-inflammatory cytokines and chemokines. One participant did not have quantifiable soluble immune factors and was excluded from the following analyses. Using multiple linear regression to control for *Gardnerella* abundance, we first observed that total urethral IgA concentration was independently associated with increased urethral IL-8 and MIP-1β (Table 2; *β* = 0.486 and *β* = 0.717 respectively, both *p* < 0.01), and a trend towards increased IL-1β (*β* = 0.475, *p* = 0.055). In contrast, *G. vaginalis*-binding IgA was not associated with the concentration of pro-inflammatory cytokines (IL-1α or IL-1β) or chemokines (IL-8 and MIP-1β). While both total IgA and *G. vaginalis-*binding IgA correlated with increased levels of the epithelial protease MMP-9 [28] (*β* = 0.870 and *β* = 0.546 respectively, both *p* < 0.05), the collinearity of *G. vaginalis-*binding IgA and total IgA meant that we could not define potential causality. Total urethral IgG concentration was not correlated with any soluble immune factors and only correlated with sE-cad (*β* = 0.531, *p* < 0.001), a marker of epithelial disruption [22]. Similar associations were observed when participants infected by *C. trachomatis* were excluded. *S. mitis*-binding IgA and IgG were strongly colinear with total IgA and IgG, respectively, (IgA: *r* = 0.502 *p* = 0.001; IgG: *r* = 0.539 *p* < 0.0001) and demonstrated similar immune associations (Supplementary Table 1).

**Table 2:** Soluble immune factors associated with total and *G. vaginalis*-binding IgA and IgG (*n* = 44)

	*G. vaginalis-*binding IgA	*G. vaginalis-*binding IgG	Total IgA	Total IgG
sE-cad	− 0.045	− 0.075	0.195	0.531***
MMP-9	0.574*	0.209	0.870***	0.170
IL-1α	0.163	0.071	0.292	− 0.034
IL-1β	0.010	− 0.014	0.475	0.284
IL-8	0.084	− 0.010	0.486**	0.244
MIP-1β	0.172	− 0.022	0.702**	0.200

β -coefficients for multiple linear regression models controlling for Gardnerella abundance. *p<0.05, **p<0.01, ***p<0.001

Using linear regression, we found that a reduced time since the last vaginal sex was correlated with both elevated urethral cytokines (IL-8: *β* = − 0.215, *p* = 0.0074; MIP-1β: *β* = − 0.1963, *p* = 0.03) and with increased concentrations of total IgA. We then performed mediation analyses to assess whether the association of time since the last vaginal sex with IL-8 and MIP-1β was mediated through total IgA. The association of time since last vaginal sex with IL-8 was partially mediated by the total IgA concentration (36.8% mediation, average casual mediation effect: − 0.079 (95% CI: − 0.137 to − 0.020), *p* = 0.012). In contrast, the association of time since last vaginal sex with MIP-1β was more substantially mediated (52.7%) by total IgA (average casual mediation effect: − 0.1035 (95% CI: − 0.1882 to − 0.04), *p* < 0.0001), such that the association between MIP-1β with time since last vaginal sex was no longer significant when controlling for total IgA (− 0.093 (95% CI: − 0.3089 to − 0.03), *p* = 0.160).

## Discussion

The urethra is a critical site of HIV acquisition in both circumcised and uncircumcised heterosexual men, and analogous to the coronal sulcus and vagina, it is likely that local inflammation in the urethra enhances HIV susceptibility [2, 3]. The urethra harbors abundant antibody-producing plasma cells that can induce local immune responses. While interactions between urethral IgA/IgG, the urethral microbiota, and local inflammation have not been previously explored, in the FGT there is evidence that vaginal IgA and IgG play a role in modulating total bacterial abundance and local inflammation [20, 21]. In the current study, we demonstrate that *G. vaginalis*-binding IgA is common in the penile urethra and is increased in the context of detectable urethral *Gardnerella* and a prior history of penile-vaginal sex, while neither the presence nor the abundance of the ubiquitous urethral species *S. mitis* was associated with *S. mitis-*binding IgA or IgG. A short duration since the last vaginal sex was strongly associated with higher urethral concentrations of both total and *G. vaginalis*-binding IgA and with elevated levels of the chemokines IL-8 and MIP-1β. These findings suggest that enrichment of *Gardnerella* in the penile urethra following sex induces a local *G. vaginalis*-binding IgA response.

While the urethral concentration of total IgA did not vary based on sexual history or the presence of *Gardnerella*, *G. vaginalis-*binding IgA was increased among participants with detectable urethral *Gardnerella*, all of whom reported a prior history of penile-vaginal sex. This suggests that *G. vaginalis*-binding IgA was induced by microbial exposure/colonization during sex and is less likely to be mediated by a non-specific, polyreactive antibody response. This contrasts with the IgA and IgG antibodies that bound the common urethral species *Streptococcus mitis*, which were strongly colinear with total antibody concentrations but did not differ based on *Streptococcus* detectability or sexual history, supporting previous observations in the gut that mucosal IgA does not have a homogenous response to all members of the microbiome [29]. These differences in mucosal host immune response may be reflective of the context in which these bacteria colonize the urethra, with *S. mitis* being a common endogenous urethral microbe, while *G. vaginalis* abundance is enriched later in life after the debut of penile-vaginal sex [9, 10].

While the immune correlates of HIV acquisition in the male urethra have not been defined in human cohort studies, elevated proinflammatory chemokines in the penile coronal sulcus (IL-8) and FGT (IL-8, MIP-1β) were independently linked with an increased risk of HIV acquisition [30, 31]. IL-8 is produced by epithelial and innate immune cells to attract neutrophils and other immune cells, and these in turn recruit highly susceptible HIV target cells such as Th17 cells [32]; MIP-1β is produced by macrophages and preferentially recruits CD4 + lymphocytes expressing the HIV co-receptor CCR5 [33]. This study shows that a shorter duration since last vaginal sex was associated with elevated concentrations of urethral IgA, in addition to prior associations to increased levels of IL-8 and MIP-1β [10], with subsequent mediation analysis demonstrating that total IgA concentration may mediate the link between recent sex and elevated chemokines. This suggests that the induction of increased urethral IgA levels by penile-vaginal sex may play a role in enhancing HIV transmission; the cause of this IgA increase is not clear, although one might hypothesize that exposure to vaginally-derived bacteria during sex triggers this response, as both *G. vaginalis*-binding IgA and total IgA were elevated in the context of recent sex.

In contrast to the FGT, where microbe-binding antibodies and total antibody concentrations were associated with reduced bacterial abundance, the penile urethra showed no inverse correlation between total IgA/IgG or *G. vaginalis*-binding IgG and total bacterial abundance. Instead, there was a positive correlation between *G. vaginalis*-binding IgA and total bacterial abundance, suggesting that there are distinct host-microbiota interactions in the tissues of the penile urethra vs. the vagina. Important contributors to these differences may include distinct environmental parameters in the vagina and penile urethra, such as frequent flushing of the urethra during urination, and the timing of specific host-bacteria interactions, with *Gardnerella* exposure potentially delayed until sexual debut.

There are important limitations to our study that merit discussion. First, we could only assess the microbe-binding IgA and IgG responses to two bacterial targets due to the limited volume of secretions obtained from a single urethral swab and the practical barriers to collecting multiple urethral swabs. Therefore, we could not quantify urethral antibody responses to other endogenous and/or potential FGT-derived species. Second, we performed 16S rRNA sequencing rather than metagenomic sequencing, and so are not able to evaluate associations of non-bacterial urethral microbiome components with the host immune response. Third, we only assessed urethral antibody responses to one species of *Streptococcus* and *Gardnerella*, and it is possible that *S. mitis* and *G. vaginalis* did not represent the dominant species in our cohort. While *Gardnerella* and *Streptococcus* species have highly conserved 16S rRNA sequences which makes it difficult to distinguish species using 16S rRNA sequencing alone, Toh et al. demonstrated that *G. vaginalis* and *S. mitis* were the two dominant species in the urethral microbiome using metagenomic shotgun sequencing and *G. vaginalis*-specific qPCR targeting the *cpn60* gene [9]. Additionally, the *G. vaginalis* (ATCC 14019) strain used in our assay was derived from a urogenital sample, while the *S. mitis* (NCTC 12261) strain is generally similar to other strains commonly found in the urogenital tract. Importantly, our data show quantifiable levels of IgA and IgG responses against these species, with unique associations tied to their prevalence. An inherent limitation of our assay is that antibodies that have already bound to endogenous bacteria in the clinical sample would not be present for quantification in the analyzed supernatant, as noted in previous studies [21]. Finally, while the low number of participants with no prior history of penile-vaginal sex limited the power of our analyses regarding sexually naïve participants, the significant findings suggest a strong biological effect. Nonetheless, additional longitudinal studies with a larger sample size of sexually inexperienced participants would help to confirm and expand on these observations.

## Conclusions

Microbes transferred during penile-vaginal sex may induce host immune responses in the male urethral with potential implications for HIV acquisition. We demonstrate that *G. vaginalis-*binding IgA is elevated among participants with a prior history of penile-vaginal sex and urethral *Gardnerella.* A short duration since the last vaginal sex was correlated with elevated *G. vaginalis*-binding IgA and total IgA concentration, with the latter independently associated with pro-inflammatory chemokines previously linked to HIV acquisition at other genital sites. These findings enhance our understanding of the interaction between the host immune system and the genital microbiota and suggest that some bacterial microbiome components may be potential targets for future vaccines.

## Supplementary Information

Supplementary Material 1: Table S1 Associations between soluble immune factors and *S. mitis-*binding IgA and IgG (*n*=40). Supplementary Figure 1. Urethral microbiome composition in uncircumcised Ugandan men. Stacked bar graph showing the relative abundance of the 20 most abundant taxa in the penile urethral microbiome, arranged in descending order of *Gardnerella* relative abundance.

## Data Availability

Sequence data for this study can be accessed at SRA project number PRJNA738496. Details for the bioinformatics analyses can be found at https://github.com/araclab/mb_analysis. Please contact author for additional requests.

## Abbreviations

AUC: Area under the curve
FGT: Female genital tract
HIV: Human immunodeficiency virus
Ig: Immunoglobulin
IgA: Immunoglobulin A
IgG: Immunoglobulin G
IL: Interleukin
MGT: Male genital tract
MIP: Macrophage inflammatory protein
MMP: Matrix metalloproteinase
