# Supplementary material for: Gardnerella vaginalis-binding IgA in the urethra of sexually experienced males
Source: Microbiome. 2025 Jan 29;13:29. doi: 10.1186/s40168-024-02007-4 (PMC11776119; doi:10.1186/s40168-024-02007-4)
Supplement: Supplementary file 2 — Supplementary Material 1: Table S1 Associations between soluble immune factors and S. mitis-binding IgA and IgG (n=40). Supplementary Figure 1. Urethral microbiome composition in uncircumcised Ugandan men. Stacked bar graph showing the relative abundance of the 20 most abundant taxa in the penile urethral microbiome, arranged in descending order of Gardnerella relative abundance. [file 40168_2024_2007_MOESM1_ESM.docx]

**Supplementary Tables:**

**Table 1. Associations between soluble immune factors and *S. mitis-*binding IgA and IgG (n=40)**

|  | *S. mitis-*binding IgA | *S. mitis-*binding IgG |
| --- | --- | --- |
| sE-cad | 0.102 | 0.234* |
| MMP-9 | 0.424* | -0.021 |
| IL-1α | 0.201 | 0.113 |
| IL-1β | 0.216 | 0.243 |
| IL-8 | 0.222 | 0.158 |
| MIP-1β | 0.394* | 0.187 |

*β -coefficients for multiple linear regression models controlling for Gardnerella abundance. *p<0.05, **p<0.01, ***p<0.001, ****p<0.0001*.

**Supplementary Figures**

**
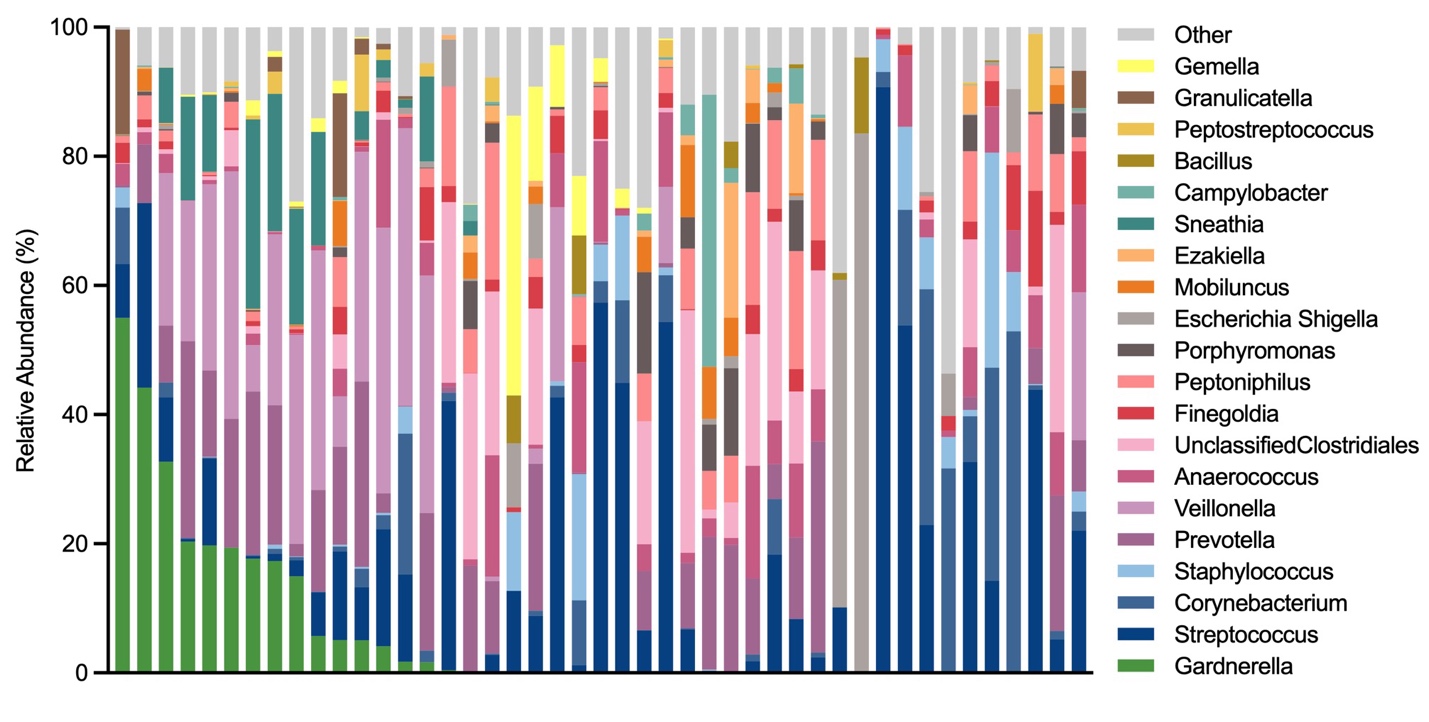
**

**Supplementary Figure 1. Urethral microbiome composition in uncircumcised Ugandan men.** Stacked bar graph showing the relative abundance of the 20 most abundant taxa in the penile urethral microbiome, arranged in descending order of *Gardnerella* relative abundance.
